# Detection and genetic characterization of circoviruses in more than 80 bat species from eight countries on four continents

**DOI:** 10.1007/s11259-023-10111-3

**Published:** 2023-03-31

**Authors:** Márton Z. Vidovszky, Szilvia Kapitány, Ákos Gellért, Balázs Harrach, Tamás Görföl, Sándor A. Boldogh, Claudia Kohl, Gudrun Wibbelt, Kristin Mühldorfer, Gábor Kemenesi, Guy-Crispin Gembu, Alexandre Hassanin, Vuong Tan Tu, Péter Estók, Anna Horváth, Győző L. Kaján

**Affiliations:** 1 Veterinary Medical Research Institute, Budapest, Hungary; 2 National Laboratory of Virology, University of Pécs, Pécs, Hungary; 3 Aggtelek National Park Directorate, Jósvafő, Hungary; 4 Centre for Biological Threats and Special Pathogens, Robert Koch Institute, Berlin, Germany; 5 Department of Wildlife Diseases, Leibniz Institute for Zoo and Wildlife Research, Berlin, Germany; 6 Faculté des Sciences, Université de Kisangani, Kisangani, République Démocratique du Congo; 7 Institut de Systématique, Évolution, Biodiversité (ISYEB), Sorbonne Université, MNHN, CNRS, EPHE, UA, Paris, France; 8 Institute of Ecology and Biological Resources, Vietnam Academy of Science and Technology, Hanoi, Vietnam; 9 Graduate University of Science and Technology, Vietnam Academy of Science and Technology, Hanoi, Vietnam; 10 Department of Zoology, Eszterházy Károly Catholic University, Eger, Hungary; 11 QUIRÓN, Center for Equine Assisted Interventions and Training for Well-Being and Sustainability, Comitán de Domínguez, Mexico

**Keywords:** Bat, Circovirus, *Cirlivirales*, Molecular typing, Screening

## Abstract

Several bat-associated circoviruses and circular rep-encoding single-stranded DNA (CRESS DNA) viruses have been described, but the exact diversity and host species of these viruses are often unknown. Our goal was to describe the diversity of bat-associated circoviruses and cirliviruses, thus, 424 bat samples from more than 80 species were collected on four continents. The samples were screened for circoviruses using PCR and the resulting amino acid sequences were subjected to phylogenetic analysis. The majority of bat strains were classified in the genus *Circovirus* and some strains in the genus *Cyclovirus* and the clades CRESS1 and CRESS3. Some strains, however, could only be classified at the taxonomic level of the order and were not classified in any of the accepted or proposed clades. In the family *Circoviridae*, 71 new species have been predicted*.* This screening of bat samples revealed a great diversity of circoviruses and cirliviruses. These studies underline the importance of the discovery and description of new cirliviruses and the need to establish new species and families in the order *Cirlivirales.*

**Supplementary Information:**

The online version contains supplementary material available at 10.1007/s11259-023-10111-3.

## Introduction

Circoviruses (family *Circoviridae*) are among the smallest viruses, and their single-stranded circular DNA genome consists of two genes: one encoding a capsid protein (Cap) and the other a replication-associated protein (Rep) (Breitbart et al. 2017). Economically important circoviruses are the beak and feather disease virus, which causes beak, claw and feather abnormalities in parrots (Fogell et al. 2016), and porcine circovirus 2 (PCV-2), which causes post-weaning multisystemic wasting syndrome (PMWS) in pigs (Segalés and Sibila 2022). The family *Circoviridae*, comprising two genera (*Circovirus* and *Cyclovirus*), has recently been classified into the order *Cirlivirales*, class *Arfiviricetes*, phylum *Cressdnaviricota*, kingdom *Shotokuvirae*, realm *Monodnaviria* (Krupovic et al. 2020). The members of the above phylum, circular Rep-encoding single-stranded DNA (CRESS DNA) viruses, have a similar genome structure to circoviruses and infect a wide range of eukaryotic hosts from unicellular organisms to plants and animals (Krupovic et al. 2020; Krupovic and Varsani 2022).

With over 1400 known species, bats (order Chiroptera) are found throughout the world excepting polar regions, extreme deserts, and a few remote islets (Simmons and Cirranello 2022). Bats are natural reservoirs for a variety of viruses, many of which are responsible for infectious diseases, such as rabies and Ebola hemorrhagic fever (Markotter et al. 2020; Gentles et al. 2020), and are putative sources of many other human and animal viruses, such as severe acute respiratory syndrome coronaviruses or canine and equine adenoviruses (Harrach et al. 2019; Hon et al. 2008; Vidovszky et al. 2015). Several bat-associated circoviruses and CRESS DNA viruses have been described, but the exact diversity and host species of these viruses are generally unknown (Dhandapani et al. 2021; Ge et al. 2011; Han et al. 2017; Hardmeier et al. 2021; Kemenesi et al. 2018; Lecis et al. 2020; Lima et al. 2015; Male et al. 2016; Matsumoto et al. 2019; Šimić et al. 2019, 2020; Zhu et al. 2018).

In this study, our aim was to describe the diversity of bat-related circoviruses and to classify the available strains according to current virus taxonomy. We therefore collected samples from more than 80 bat species in eight countries on four continents and tested them for circoviruses. The resulting viral sequences were subjected to phylogenetic analysis together with reference sequences and other bat circoviruses. Based on preliminary analyses, we also detected bat associated cirliviruses that were not classified in the family *Circoviridae*, thus extending the scope of the phylogenetic analysis to the order *Cirlivirales.*

The typing of strains was based on partial Rep sequences, but the official species demarcation criteria for both circoviruses and cycloviruses are based on the whole genome sequence (Breitbart et al. 2017). Therefore, a new method based on partial Rep sequence identity was developed and strains were classified in the accepted virus species using this method. Using the same method, we also established hypothetical novel virus species whose classification should be confirmed in the future based on complete genome sequences.

## Materials and methods

### Origin of bat samples and DNA extraction

Expeditions were carried out in Namibia (Erongo and Kunene) in 2012, in the Democratic Republic of the Congo (Tshopo) in 2013, in Vietnam (Thanh Hoa and Cao Bang) in 2014 and in Mexico (Chiapas) in 2015. In Europe, samples were collected in Germany, Hungary, Romania, and Slovakia. In total, 424 bat samples from 408 bats or colonies of more than 80 species were collected in eight countries on four continents. These were fecal samples collected from live individuals during mist netting (*n* = 268) or as guano from under colonies (*n* = 56), internal organs (heart, liver, intestine, kidney, brain, spleen and salivary gland) from dead bats (*n* = 97), urine (*n* = 2) or anal swabs (*n* = 1). The guano samples were collected almost exclusively in buildings, where homogenous, single-species colonies tend to occur. Sample collectors took the utmost care and collected guano samples only from colonies where bats could be identified. Exceptions were colonies of *Myotis myotis*, where some *Myotis blythii* bats may have been present, so in this case the species pair *Myotis myotis*/*blythii* was used to designate the host species. The sources of samples are summarized in Table 1. DNA was extracted from the fecal samples using the E.Z.N.A. DNA Stool Kit (OMEGA Bio-Tek) and from the other samples using the NucleoSpin Tissue Kit (Macherey–Nagel).

**Table 1:** Analyzed bat samples

	Species name		Location	Type	Number of	
	Scientific	Common			Sampled animal	Positive
	**Suborder Yinpterochiroptera**					
	**Superfamily Pteropodoidae**					
	**Family Pteropodidae**					
1	*Casinycteris argynnis*	short-palated fruit bat	DRC	f	4	1
2	*Cynopterus* cf.* sphinx*	greater short-nosed fruit bat	Vietnam	f	1	
3	*Epomops franqueti*	Franquet's epauletted fruit bat	DRC	f	5	2
4	*Hypsignathus monstrosus*	hammer headed bat	DRC	f	4	
5	*Megaloglossus woermanni*	Woermann's bat	DRC	f	2	
6	*Myonycteris torquata*	little collared fruit bat	DRC	f	4	1
7	*Pteropus giganteus*	Indian flying fox	captivity	g	1	1
8	*Pteropus lylei*	Lyle's flying fox	captivity	o	3 (7)	
9	*Rousettus aegyptiacus*	Egyptian fruit bat	captivity	g	1	
				o	4 (12)	
10	*Scotonycteris zenkeri*	Zenker's fruit bat	DRC	f	1	
	**Superfamily Rhinolophoidae**					
	**Family Hipposideridae**					
11	*Aselliscus stoliczkanus*	Stoliczka's trident bat	Vietnam	f	1	
12	*Hipposideros armiger*	great roundleaf bat	Vietnam	f	1	1
13	*Hipposideros caffer*	Sundevall's roundleaf bat	Namibia	f	1	
	*Hipposideros* cf. *caffer*		DRC	f	2	
14	*Hipposideros fuliginosus*	sooty roundleaf bat	DRC	f	1	1
15	*Hipposideros* cf. *gigas*	giant roundleaf bat	DRC	f	1	
	*Hipposideros* sp.		DRC	f	18	5
	*Hipposideros* spp.		DRC	g	1	
	**Family Rhinolophidae**					
16	*Rhinolophus affinis*	intermediate horseshoe bat	Vietnam	f	9	1
17	*Rhinolophus euryale*	Mediterranean horseshoe bat	Hungary	g	3	
18	*Rhinolophus ferrumequinum*	greater horseshoe bat	Hungary	f	1	
			Hungary	g	6	1
19	*Rhinolophus hipposideros*	lesser horseshoe bat	Hungary	g	12	3
20	*Rhinolophus episcopus*	Allen’s horseshoe bat	Vietnam	f	1	1
21	*Rhinolophus pearsonii*	Pearson's horseshoe bat	Vietnam	f	2	
22	*Rhinolophus pusillus*	least horseshoe bat	Vietnam	f	4	
	*Rhinolophus cf. pusillus*	least horseshoe bat	Vietnam	f	1	
23	*Rhinolophus rex*	king horseshoe bat	Vietnam	f	1	
24	*Rhinolophus thomasi*	Thomas's horseshoe bat	Vietnam	f	1	
	**Suborder Yangochiroptera**					
	**Superfamily Emballonuroidae**					
	**Family Nycteridae**					
25	*Nycteris* cf. *arge*	Bates's slit-faced bat	DRC	f	2	
26	*Nycteris grandis*	large slit-faced bat	DRC	f	4	1
27	*Nycteris* cf. *hispida*	hairy slit-faced bat	DRC	f	4	1
	*Nycteris* sp.		Namibia	f	1	
	**Superfamily Noctilionoidae**					
	**Family Mormoopidae**					
28	*Mormoops megalophylla*	ghost-faced bat	Mexico	f	1	
29	*Pteronotus gymnonotus*	big naked-backed bat	Mexico	f	2	
30	*Pteronotus parnelli*	Parnell's mustached bat	Mexico	f	3	
	**Family Phyllostomidae**					
31	*Anoura geoffroyi*	Geoffroy's tailless bat	Mexico	f	2	
32	*Artibeus jamaicensis*	Jamaican fruit bat	Mexico	f	3	
33	*Carollia sowelli*	Sowell's short-tailed bat	Mexico	f	3	
34	*Glossophaga soricina*	Pallas's long-tongued bat	Mexico	f	1	
35	*Phyllostomus discolor*	pale spear-nosed bat	captivity	o	1 (3)	
36	*Sturnira lilium*	little yellow-shouldered bat	Mexico	f	1	
	**Superfamily Vespertilionoidae**					
	**Family Miniopteridae**					
37	*Miniopterus schreibersii*	common bent-wing bat	Hungary	f	5	
			Slovakia	f	1	
	**Family Molossidae**					
38	*Chaerephon* cf. *pumilus*	little free-tailed bat	DRC	f	1	
	*Chaerephon* sp.		DRC	f	1	
	*Molossidae* sp*.*		DRC	g	1	
39	*Mops* cf. *midas*	Midas free-tailed bat	DRC	f	1	1
40	*Sauromys petrophilus*	Roberts's flat-headed bat	Namibia	f	1	
41	*Tadarida brasiliensis*	Mexican free-tailed bat	Mexico	f	1	
	**Family Natalidae**					
42	*Natalus mexicanus*	Mexican greater funnel-eared bat	Mexico	f	3	
	**Family Vespertilionidae**					
43	*Barbastella barbastellus*	western barbastelle	Hungary	f	5	1
			Romania	f	2	1
44	*Eptesicus nilssonii*	northern bat	Germany	o	1	
			Hungary	f	1	1
45	*Eptesicus serotinus*	serotine bat	Hungary	f	4	1
			Hungary	g	10	1
			Hungary	u	1	
			Slovakia	f	5	1
			Germany	o	1	
46	*Glauconycteris argentata*	silvered bat	DRC	f	1	
	*Glauconycteris* cf. *argentata*		DRC	f	3	
47	*Glauconycteris beatrix*	Beatrix's bat	DRC	f	5	
48	*Glauconycteris superba*	pied butterfly bat	DRC	f	4	3
49	*Hypsugo cadornae*	Cadorna's pipistrelle	Vietnam	f	1	
50	*Hypsugo savii*	Savi's pipistrelle	Hungary	f	1	
51	*Kerivoula furva*	dark woolly bat	Vietnam	f	3	1
52	*Myotis alcathoe*	alcathoe bat	Hungary	f	5	
			Hungary	u	1	
			Romania	f	2	1
53	*Myotis* cf. *alticraniatus*	Indochinese whiskered myotis	Vietnam	f	1	
54	*Myotis bechsteinii*	Bechstein's bat	Hungary		15	1
			Romania		2	
55	*Myotis blythii*	lesser mouse-eared bat	Romania	f	1	
			Hungary	as	1	
			Hungary	f	1	
56	*Myotis bocagii*	rufous mouse-eared bat	DRC	f	2	
57	*Myotis brandtii*	Brandt's bat	Hungary	f	4	
			Romania	f	2	
58	*Myotis dasycneme*	pond bat	Hungary	f	4	
			Hungary	g	1	
59	*Myotis daubentonii*	Daubenton's bat	Hungary	f	12	3
			Hungary	g	1	
			Germany	o	1	
60	*Myotis emarginatus*	Geoffroy's bat	Hungary	g	2	
61	*Myotis* cf. *muricola*	wall-roosting mouse-eared bat	Vietnam	f	1	
62	*Myotis myotis*	greater mouse-eared bat	Hungary	f	3	
			Romania	f	2	
	*Myotis myotis/blythii*	greater/lesser mouse-eared bat	Hungary	g	9 (10)	3 (4)
63	*Myotis mystacinus*	whiskered bat	Hungary	f	2	
			Germany	o	4	1
64	*Myotis nattereri*	Natterer's bat	Hungary	f	7	
65	*Myotis pilosatibialis*	northern hairy-legged myotis	Mexico	f	2	
66	*Myotis sicarius*	Mandelli’s mouse-eared bat	Vietnam	f	1	
	*Myotis* spp.		Mexico	f	14	1
	*Neoromicia* spp.		DRC	f	4	1
67	*Neoromicia zuluensis*	Zulu serotine	Namibia	f	1	
68	*Nyctalus leisleri*	lesser noctule	Germany	o	1	1
			Hungary	f	1	
			Slovakia	f	3	
69	*Nyctalus noctula*	common noctule	Germany	o	42	3
			Hungary	f	6	1
			Hungary	g	1	
	*Phoniscus* sp.		Vietnam	f	1	1
70	*Pipistrellus abramus*	Japanese pipistrelle	Vietnam	f	2	
71	*Pipistrellus coromandra*	Indian pipistrelle	Vietnam	f	2	
72	*Pipistrellus javanicus*	Java pipistrelle	Vietnam	f	1	
73	*Pipistrellus kuhlii*	Kuhl's pipistrelle	Germany	o	1	
74	*Pipistrellus nathusii*	Nathusius' pipistrelle	Germany	o	4	
75	*Pipistrellus pipistrellus*	common pipistrelle	Hungary	f	3	
			Hungary	g	1	
			Germany	o	10	1
76	*Pipistrellus pygmaeus*	soprano pipistrelle	Hungary	f	2	
			Hungary	g	1	
			Germany	o	1	
77	*Pipistrellus tenuis*	least pipistrelle	Vietnam	f	1	
	*Pipistrellus* sp.		DRC	f	4	
78	*Plecotus auritus*	brown long-eared bat	Hungary	f	5	
			Slovakia	f	1	
			Germany	o	2	
79	*Plecotus austriacus*	grey long-eared bat	Hungary	f	1	
			Hungary	g	1	
80	*Scotophilus dinganii*	African yellow bat	DRC	f	1	
81	*Vansonia rueppellii*	Rüppell’s bat	DRC	f	1	
82	*Vespertilio murinus*	parti-coloured bat	Hungary	o	1 (2)	1 (2)
			Germany	o	5	1
	Chiroptera sp.	bat	DRC	f	6	1
	SUM				408 (424)	52 (54)

If multiple samples were investigated from the same animal(s), sample numbers are indicated in brackets

Abbreviations: *DRC* Democratic Republic of the Congo; *as* anal swab (from live caught bat), *f* feces (from live caught bat); *g* guano (under bat colony), *o* organ (from moribund or dead animal), *u* urine (from live caught bat)

### PCR and sequencing

Samples were tested for circoviruses using nested PCRs targeting the Rep coding gene: first, the PCR developed by Halami was used (Halami et al. 2008), and later the one developed by Li et al. (2010). The PCR products of the second round were sequenced using the Sanger method and the sequences were deposited in the NCBI Nucleotide database under accession numbers OP380746-OP380799.

### Phylogenetic analyses

The sequences were assembled and translated into amino acid sequences using Geneious. To facilitate proper phylogenetic placement, where available, the full Rep amino acid sequences of the reference strains were used and aligned with the partial coding sequences to infer the tree. The strains were first typed in a preliminary phylogenetic tree reconstruction: the entire class *Arfiviricetes* was represented, and here all the virus strains we analysed were clustered into the order *Cirlivirales.* Thus, the second analysis focused only on this order. The reference sequences were derived from the ICTV Virus Metadata Resource v37.2 database and were supplemented with CRESS1–5 sequences based on the publication of Kazlauskas et al. (2019). An additional 195 bat-associated cirlivirus strains were included in the analyses (Dhandapani et al. 2021; Ge et al. 2011; Han et al. 2017; Hardmeier et al. 2021; Kemenesi et al. 2018; Lecis et al. 2020; Lima et al. 2015; Male et al. 2016; Matsumoto et al. 2019; Šimić et al. 2019, 2020; Zhu et al. 2018).

The amino acid sequences were aligned using the MAFFT E-INS-i algorithm (Katoh and Standley 2013). The length of the edited alignment was 333 amino acids for the first alignment and 325 amino acids for the second. ModelTest-NG v0.1.6 proposed the use of the evolutionary model developed by Le and Gascuel (LG) with invariant site ratios (+ I) and discrete Gamma-rate categories (+ G) for the reconstruction of both phylogenetic trees (Darriba et al. 2020; Le and Gascuel 2008). The best phylogenetic tree was selected from 300 replicates inferred using RAxML-NG v1.1.0, and the robustness of the tree was determined by a non-parametric bootstrap calculation with 1000 replicates for cirliviruses (Kozlov et al. 2019). For this tree, we applied transfer bootstrap expectation values (Lemoine et al. 2018). Phylogenetic trees were visualized using MEGA 7 and rooted at the midpoint with only bootstrap values ≥ 75% at the nodes (Kumar et al. 2016).

The circovirus strains were compared to the reference strains of all officially accepted circovirus species: to quantify the evolutionary distance, pairwise amino acid sequence identity analysis was performed on the basis of partial Rep sequences using the Sequence Demarcation Tool v1.2. The analyzed portion of the Rep proteins was homologous to the 55 to 198 amino acid segment of the Rep of the porcine circovirus 1 (PCV-1) reference strain (AAC34819).

### Whole genome sequencing and protein modeling

As mentioned in the introduction, new species in the family *Circoviridae* are delimited based on whole genome sequence identity (Breitbart et al. 2017). Although we did not yet have a complete genome sequence, based on the partial Rep sequence, strain GT757B showed low amino acid sequence identity to reference strains of circoviral species already accepted at the time, and we therefore hypothesized that this strain may represent a new candidate species. Therefore, we performed whole genome sequencing on it: we used inverted primers to amplify the whole genome and performed primer walking and Sanger sequencing on this amplified segment. The primers used are listed in Table S1. The complete genome sequence was compared with the genome sequence of all circoviruses using the Sequence Demarcation Tool v1.2.

The initial methionine amino acid of the Cap protein was replaced with serine in strain GT757B, so, to compare Cap structures, the complete protein was modeled from both strain GT757B and strain Acheng30 (ASU92176), both of which belong to species *Bat associated circovirus 10,* using AlphaFold2 (Jumper et al. 2021). For each protein, five models were generated and scored using the protein quality assignment module of AlphaFold2, and the best one was used for visualization (Schrödinger 2021). Molecular graphics were generated using VMD version 1.9.3 (Humphrey et al. 1996).

## Results

### Virus presence

Of the 424 bat samples tested, 54 (13%) were positive for cirlivirus. The positivity rate for tissue samples was 9%, for individually collected fecal samples 13% and for guano samples 20%. Among the different bat families, the highest positivity rate was measured in the family Hipposideridae (27% of 26 samples tested), while no cirlivirus was detected in the families Mormoopidae (*n* = 6), Natalidae (*n* = 3) and Phyllostomidae (*n* = 14). Virus presence in the samples is summarized in Table 1.

### Phylogenetic analysis

**Fig. 1:**
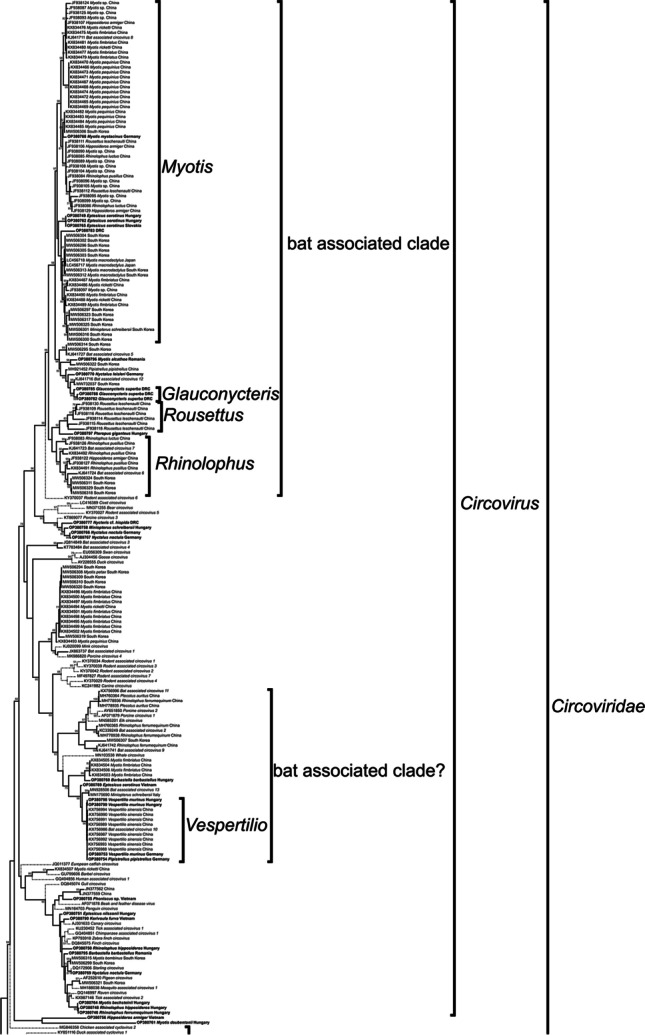
Fragment of the phylogenetic tree based on bat cirlivirus Rep amino acid sequences, showing the genus *Circovirus*. The complete phylogenetic tree is shown in Fig. S1. Strains are indicated by their nucleotide accession number, host species and country of collection (if available). Newly detected virus strains are in bold, and branches of cirliviruses not associated with bats are shown in dashed lines. Abbreviation: DRC, Democratic Republic of the Congo

**Fig. 2:**
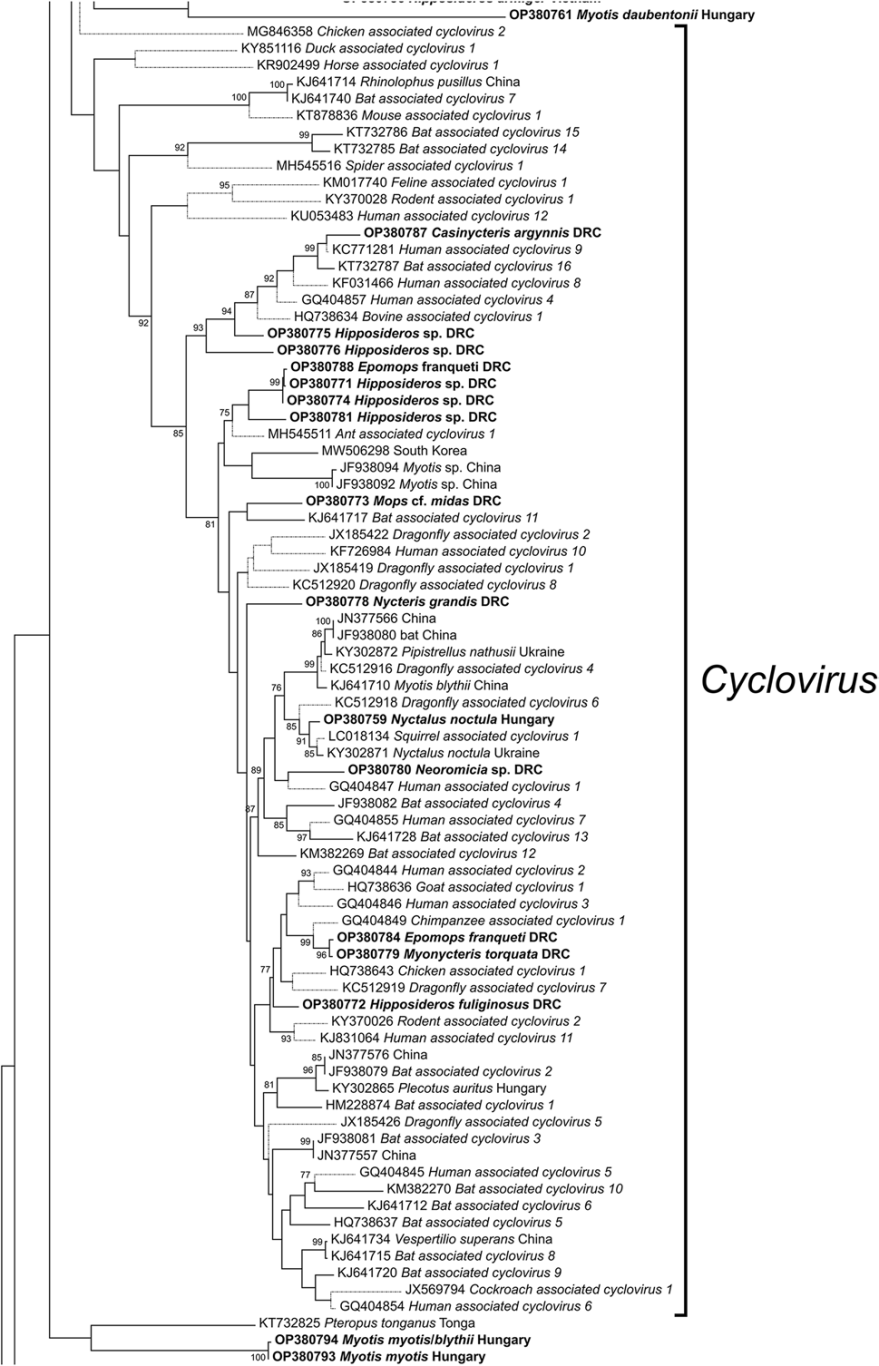
Fragment of the phylogenetic tree based on bat cirlivirus Rep amino acid sequences, showing the genus *Cyclovirus*. The complete phylogenetic tree is shown in Fig. S1. Strains are indicated by their nucleotide accession number, host species and country of collection (if available). Newly detected virus strains are in bold, and branches of cirliviruses not associated with bats are shown in dashed lines. Abbreviation: DRC, Democratic Republic of the Congo

**Fig. 3:**
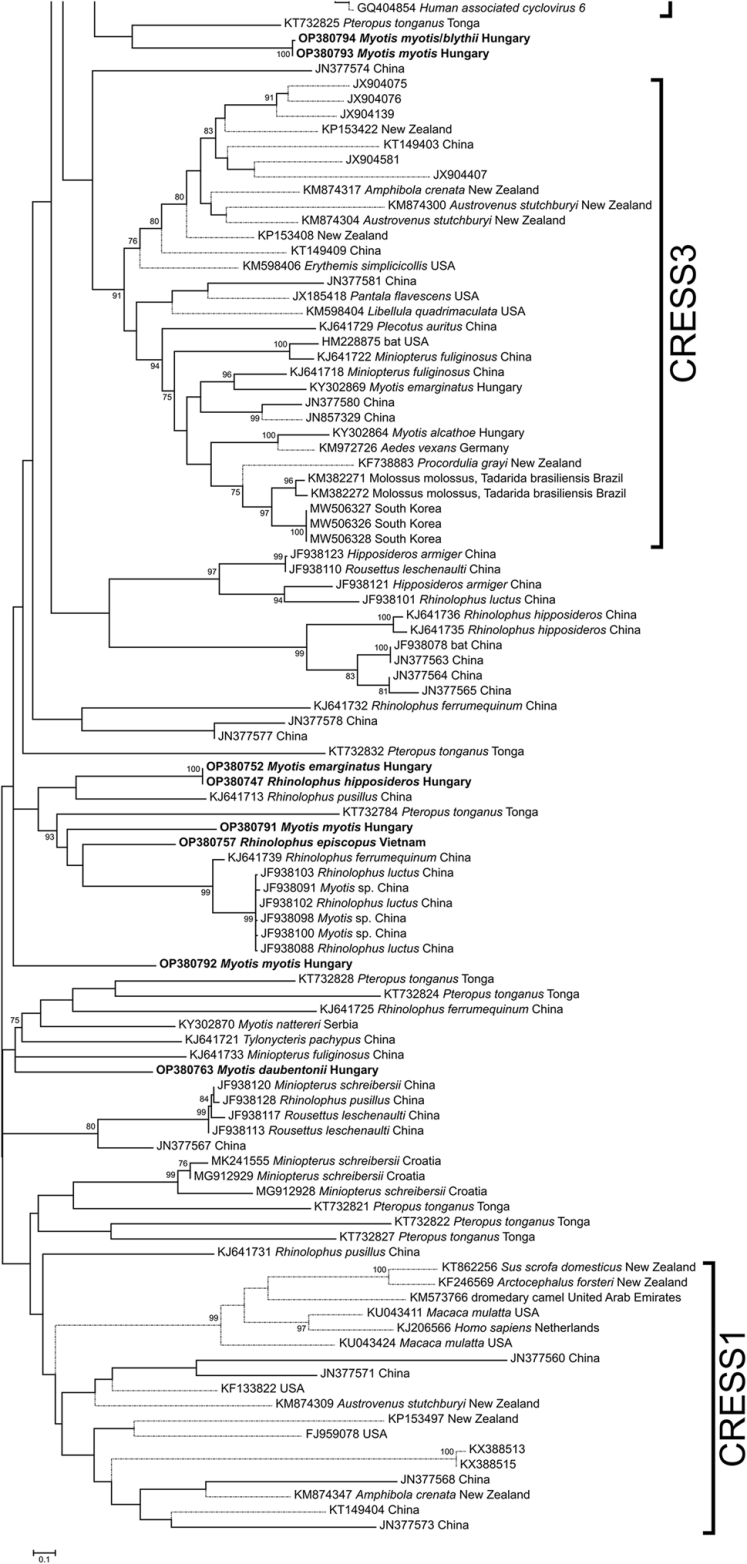
Fragment of the phylogenetic tree based on bat cirlivirus Rep amino acid sequences, showing the bat cirliviruses outside the *Circoviridae* and *Vyliaviridae* families. The complete phylogenetic tree is shown in Fig. S1. Strains are indicated by their nucleotide accession number, host species and country of collection (if available). Newly detected virus strains are in bold, and branches of cirliviruses not associated with bats are shown in dashed lines

The phylogenetic tree of the cirlivirus strains is shown in Figs. 1, 2 and 3 and S1, and that of the arfiviruses (the class *Arfiviricetes* of CRESS DNA viruses) in Fig. S2. Of the strains detected, 30 were classified in the genus *Circovirus*, 14 in the genus *Cyclovirus*, four in the family *Circoviridae* and six in the order *Cirlivirales*, but did not belong to any of the accepted or proposed clades. Of the 195 other bat cirlivirus strains, 126 were classified in the genus *Circovirus*, 13 in the genus *Cyclovirus*, one in the family *Circoviridae* but outside these two genera, four in the clade CRESS1, nine in the clade CRESS3 and 42 in the order *Cirlivirales* but not in any accepted or proposed clade. Of all bat cirlivirus strains analyzed, 38% formed a monophyletic clade exclusively with the reference strains of *Bat associated circovirus 5–8* and *12*. These virus strains originated from 16 bat species of 10 genera and three continents.

The official species demarcation criteria for circoviruses and cycloviruses are based on whole genome sequences (Breitbart et al. 2017). As these were not available for comparison, a new partial-Rep amino-acid-based threshold for species demarcation was defined. Based on the pairwise sequence identity of partial Rep-amino acid sequences of circoviruses (family *Circoviridae*), the highest sequence identity between reference strains of two accepted species was 95.17%, measured between reference strains of *Human associated cyclovirus 9* (KC771281) and *Bat associated cyclovirus 16* (KT732787) (Male et al. 2016; Smits et al. 2013). This defined provisional threshold was used to classify strains into already accepted species and to delimit datasets representing new species, in other words, clades representing novel species. As previously mentioned, this provisional threshold does not replace the formal species demarcation criteria set by the ICTV *Circoviridae* Study Group: 'species demarcation threshold is 80% genome-wide nucleotide sequence identity based on the distribution of pairwise identities' (Breitbart et al. 2017; Rosario et al. 2017). However, as complete genome sequences were not available, we classified virus strains into probable species based on this provisional threshold. Of course, mainly due to frequent recombination events (Kazlauskas et al. 2018), this species classification was probably not always accurate.

The results of the pairwise amino acid sequence identity analysis are summarized in Table S2. Of the 54 bat strains detected, five were classifiable into already accepted species and 31 datasets representing new species could be generated with the defined threshold: these datasets, in other words these clades, contained virus strains with sequence identity of at least 95.18% and sequence identity between datasets was always below this threshold. Of the 132 additional bat circovirus strains, 33 could be classified in already accepted species and 40 additional species-representing datasets (clades), not yet generated using the detected strains, were generated with the defined sequence identity threshold. These datasets contained 1–14 strains.

### Whole genome analysis

**Fig. 4:**
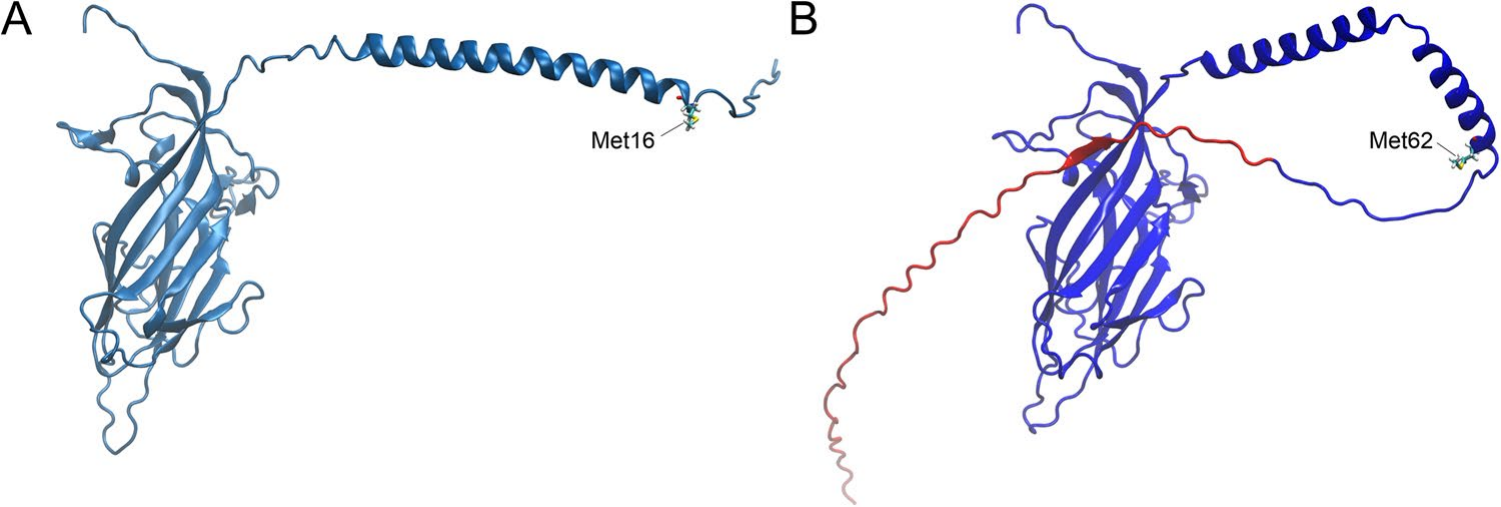
Capsid protein model of two *Bat associated circovirus 10* strains. A: Acheng30 (ASU92176) reference strain; B: GT757B strain. Homologous domains are indicated in blue, while the N-terminal stretch of the capsid protein in strain GT757B is indicated in red. The second methionine of the capsid protein of strain Acheng30 and the homologous methionine of strain GT757B are also highlighted

The whole genome sequence from strain GT757B was found to be 2117 bp long with 53.7% G + C content. A typical circovirus genome arrangement with one Rep and one Cap coding gene was detected. The highest overall genome sequence identity was measured with strain Acheng30 (KX756986), reference strain of species *Bat associated circovirus 10,* and the measured identity was 90.85%. The modeled structure of the Cap protein is visualized in Fig. 4. When the predicted Cap protein was compared to the corresponding protein of the Acheng30 strain (ASU92176), homology and relatively high amino acid sequence identity (81%) were observed. However, the deduced amino acid sequence was 45 amino acids longer or 15 amino acids shorter compared to the reference strain. Based on the protein model, the longer, extra N-terminal portion was predicted to be disordered.

## Discussion

Recently, several bat circovirus screenings have been performed (Dhandapani et al. 2021; Ge et al. 2011; Han et al. 2017; Hardmeier et al. 2021; Kemenesi et al. 2018; Lecis et al. 2020; Lima et al. 2015; Male et al. 2016; Matsumoto et al. 2019; Šimić et al. 2019, 2020; Zhu et al. 2018). In our work, we aimed to evaluate the diversity of virus strains found in our screening studies and those of others.

Our circovirus screening showed a 13% positivity rate. Similar positivity rates have been detected in bat populations from Myanmar (7%), Japan (14%), Korea (15%), Brazil (24%), and China (24% and 37%) (Dhandapani et al. 2021; Ge et al. 2011; Han et al. 2017; He et al. 2013; Lima et al. 2015; Matsumoto et al. 2019). The higher positivity rates in our guano samples compared to individual fecal and tissue samples may be explained by the presence of populous colonies: we hypothesize, that infected individuals may contaminate large amounts of guano samples with their feces or urine, even at low morbidity rates.

Since the screening of bat samples was performed for many years, some modifications in the methodology were made during this long period: the screening PCR method was changed from the PCR developed by Halami (resulting strains: OP380746–OP380752) to the one developed by Li (resulting strains: OP380753–OP380799) (Halami et al. 2008; Li et al. 2010). This decision was made to broaden the target range of the PCR system used: the former PCR targets members of the *Circovirus* genus, while the latter also targets members of the *Circoviridae* family, i.e. cycloviruses as well. Furthermore, since the amplified partial *Rep* gene sequences overlap significantly, this change did not affect the phylogenetic analysis. Both PCR systems resulted in strains typed as circoviruses, the Li system also resulted in cycloviruses, and again both resulted in cirliviruses that could not be classified into any established or proposed viral taxonomic clade. Thus the true target ranges of both PCRs are wider than originally predicted. Although the Halami PCR has been used to detect strains outside the *Circoviridae* family, the ability to detect cycloviruses remains unanswered, as no such virus strains were detected using it.

The majority of the phylogenetically analyzed strains (63%) belong to the genus *Circovirus.* Based on the large number of vertebrate circoviruses and the monophyletic placement of circoviruses from relatively closely related hosts, a relatively long coevolution is hypothesized between vertebrates and their circoviruses (Kaján et al. 2020); and based on this, it is most likely that most species of chiroptera carry circoviruses. The circoviral dominance shown in our analyses therefore seems plausible, while cyclovirus strains may be at least partly insect in origin (Rosario et al. 2012a, 2018). A high proportion of bat species are insectivorous, and almost all of the samples we examined contained either intestines or fecal matter. Although this latter hypothesis should be treated with caution, as several vertebrate-associated cycloviruses have been reported (Li et al. 2010, 2011; Tan et al. 2013; Zhang et al. 2014), in addition to several herbivorous bat associated cycloviruses (Male et al. 2016). The theory of long-term coevolution of circoviruses and bats is further supported by their diverse, monophyletic lineage within the genus *Circovirus*, which comprises 38% of all strains analyzed. Among these strains, additional subclades were identified that contained circoviruses primarily or exclusively from a single bat genus. In the largest subclade (*n* = 69), 78% of the available host species were from different Myotis species from three continents (Africa, Asia and Europe), and additional smaller clades were identified in the genus *Rhinolophus* and the species *Rousettus leschenaultii* and *Glauconycteris superba.*

However, host species analysis of another clade rich in bat-virus strains may also shed light on host-switching virus strains. Of the bat cirlivirus strains analyzed, 10% formed a monophyletic clade with the reference strains of *Bat associated circovirus 2*, *9–11,* and *13,* and reference strains of *Elk*, *Whale* and *Porcine circovirus 1* and *2*. Such localization of the porcine circoviruses supports the hypothesis of a host switch from bats (Li et al. 2019) and raises the possibility of similar origins for the elk and whale circoviruses. Viruses that have recently changed hosts tend to have increased pathogenicity in the new host because the host has not yet been able to adapt and evolve appropriate defence mechanisms (Kaján et al. 2020). These viruses have been detected from a moribund Rocky Mountain elk, and a dead Longman’s beaked whale, while PCV-2 causes PMWS in pigs (Dán et al. 2003; Fisher et al. 2020; Landrau-Giovannetti et al. 2020).

It is not possible to say with absolute certainty that bats are the true hosts of the many unclassifiable cirliviruses (19% of the strains analyzed). Despite recent significant advances in the field (Kinsella et al. 2020; Krupovic et al. 2020; Krupovic and Varsani 2022), these strains were not grouped into any accepted (e.g., *Vilyaviridae* family) or proposed (e.g., CRESS1 or CRESS3) clade within the order *Cirlivirales*. Further in-depth studies are needed in the uncharted area of CRESS DNA virus diversity to identify the true host species and to type these detected strains.

With the exception of the many reference strains, often not bat, only bat cirliviruses were analyzed and no other host species were included in the analysis, as such a phylogenetic tree would be difficult to analyse and visualize. Although it is arguable that such a broad analysis would have major advantages in i) identifying true host species, ii) coevolution analysis, and iii) in establishing and confirming new viral clades.

The typing of some strains was questionable as it did not give consistent results. For example, strain 2013–34-BS (OP380761) was clustered outside the *Circoviridae* family in the arfivirus analysis, while it was included in the cirlivirus analysis. Such inconsistencies may be caused by the limited length of the available segment or recombination events between Rep domains (Kazlauskas et al. 2018). Whole genome comparisons will provide a more accurate phylogenetic placement of these. Furthermore, phylogenetic misplacement of the *Baphyvirales*, *Recrevirales* and *Rivendellvirales* orders or a split CRESS1 clade was also observed in the arfivirus analysis (Fig. S2). These anomalies are also likely to be caused by recombination events (Kazlauskas et al. 2018). When reference strains were analyzed alone, without bat cirliviruses, the phylogenetic location of the reference strains reflected the current taxonomy (Krupovic and Varsani 2022).

The strain Tbat_H_103163 (KT732825), originally typed as Pacific flying fox faeces-associated circular DNA virus 8 (Male et al. 2016), was classified by Kazlauskas et al. (2019) in the family *Circoviridae.* Thus, two closely related bat circovirus strains were similarly typed, although in Kazlauskas' analysis and here, these strains formed a monophyletic unit with the family but were placed as an outgroup. Since the close relationship of these strains to the taxonomic family was supported, they were placed in the family. However, further classification at genus level is very uncertain. As these results may be influenced by recombination events in the Rep domain (Kazlauskas et al. 2018), whole genome sequences are needed for more accurate typing in this case as well.

Although 424 samples were screened and an additional 195 strains were typed without geographical restriction, no strains of the species *Bat associated circovirus 1*, *3*, *4*, *6* or *7* or *Bat associated cyclovirus 1*, *4–6,* or *9–15* were found. After analyzing such a large number of samples and strains, it is questionable whether the reference strains of the above species are indeed infecting bats. Perhaps a more extensive phylogenetic analysis of circoviruses from other host species could at least partially shed light on this picture. Circovirus species should be named more carefully to avoid misleading researchers in the future.

Since 80% nucleotide sequence identity measured on the whole genome was defined as the species demarcation criterion (Breitbart et al. 2017), strain GT757B did not represent a new species as it is classified as species *Bat associated circovirus 10.* Yet, based on sequence identity analysis, 71 new species were predicted in the *Circoviridae* family alone. If we accept the second highest Rep amino acid sequence identity (89.86%) between the accepted species as a threshold, the number of predicted new species was still 57. This finding, together with the large number of described bat species, predicts the existence of many additional bat circovirus and cyclovirus species.

The Cap-encoding gene of strain GT757B shows a high degree of similarity to the gene of the reference strain of the species. Yet, the product of the former is assumed to be truncated by 15—well conserved—amino acids in the N-terminal part of the protein compared to the latter, since the N-terminal extension of the protein to the previous available start codon is contradicted by several observations: i) it is an alternative start codon (CUG) (Starck et al. 2012), ii) it is beyond the origin of replication of the genome (Rosario et al. 2012b), iii) and the extra N-terminal part of the protein was predicted to be disordered. Although the predicted Cap-encoding gene of fly associated circular virus 6 (MH545530, species *Illuinvirus amon*, family *Vilyaviridae*) also extends beyond the origin of replication (Rosario et al. 2018). Thus, the true transcript length of this gene requires further investigation.

The GT757B strain was detected from a *Vespertilio murinus* that died in Hungary and belongs to a virus clade (*n* = 13) in which 92% of the hosts belong to the genus *Vespertilio.* Circoviruses from both species of the bat genus have been detected in distant areas (China, Germany, Hungary), and have shown close relationship to each other during the phylogenetic reconstruction. This observation may also suggest a relatively long-standing virus-host coevolution.

The screening work carried out revealed a high diversity of circo- and other cirliviruses in the bat samples tested. Our work has highlighted circovirus lineages coevolving with bats and possible host switches. In any case, further studies are needed on the high diversity of circoviruses and cirliviruses that cannot be classified into known species or into known virus families. These studies underline the importance of the discovery and description of new cirliviruses and the need to establish new species and families in the order *Cirlivirales.*

## Supplementary Information

Below is the link to the electronic supplementary material.

Supplementary file1 (PDF 106 KB) Fig. S1 Phylogenetic tree reconstruction of bat cirliviruses based on Rep amino acid sequences. Fragments of the same phylogenetic tree are shown in Figures 1–3. Strains are indicated by their nucleotide accession number, host species and country of collection (if available). Newly detected virus strains are in bold, and branches of cirliviruses not associated with bats are in red. Reference strains of bat associated circovirus and cyclovirus species are indicated in blue

Supplementary file2 (PDF 113 KB) Fig. S2 Phylogenetic tree reconstruction of bat cirliviruses with arfiviruses (class *Arfiviricetes*) based on Rep amino acid sequences. The strains are indicated by their nucleotide accession number, host species and country of collection (if available), except for CRESS1-5 strains, which are indicated by either nucleotide or Rep protein accession number. Newly detected virus strains are in bold, and branches of cirliviruses not associated with bats are indicated in red. The reference strains of bat associated circoviruses and cycloviruses are indicated in blue

Supplementary file3 (DOCX 13 KB)

Supplementary file4 (XLSX 17 KB)

## Data Availability

The sequences are deposited in the NCBI Nucleotide database under accession numbers OP380746-OP380799.
