# Supplementary material for: Detection and genetic characterization of circoviruses in more than 80 bat species from eight countries on four continents
Source: Vet Res Commun. 2023 Mar 31;47(3):1561–73. doi: 10.1007/s11259-023-10111-3 (PMC10066014; doi:10.1007/s11259-023-10111-3)
Supplement: Supplementary file 1 — Supplementary file1 (PDF 106 KB) Fig. S1 Phylogenetic tree reconstruction of bat cirliviruses based on Rep amino acid sequences. Fragments of the same phylogenetic tree are shown in Figures 1–3. Strains are indicated by their nucleotide accession number, host species and country of collection (if available). Newly detected virus strains are in bold, and branches of cirliviruses not associated with bats are in red. Reference strains of bat associated circovirus and cyclovirus species are indicated in blue [file 11259_2023_10111_MOESM1_ESM.pdf]

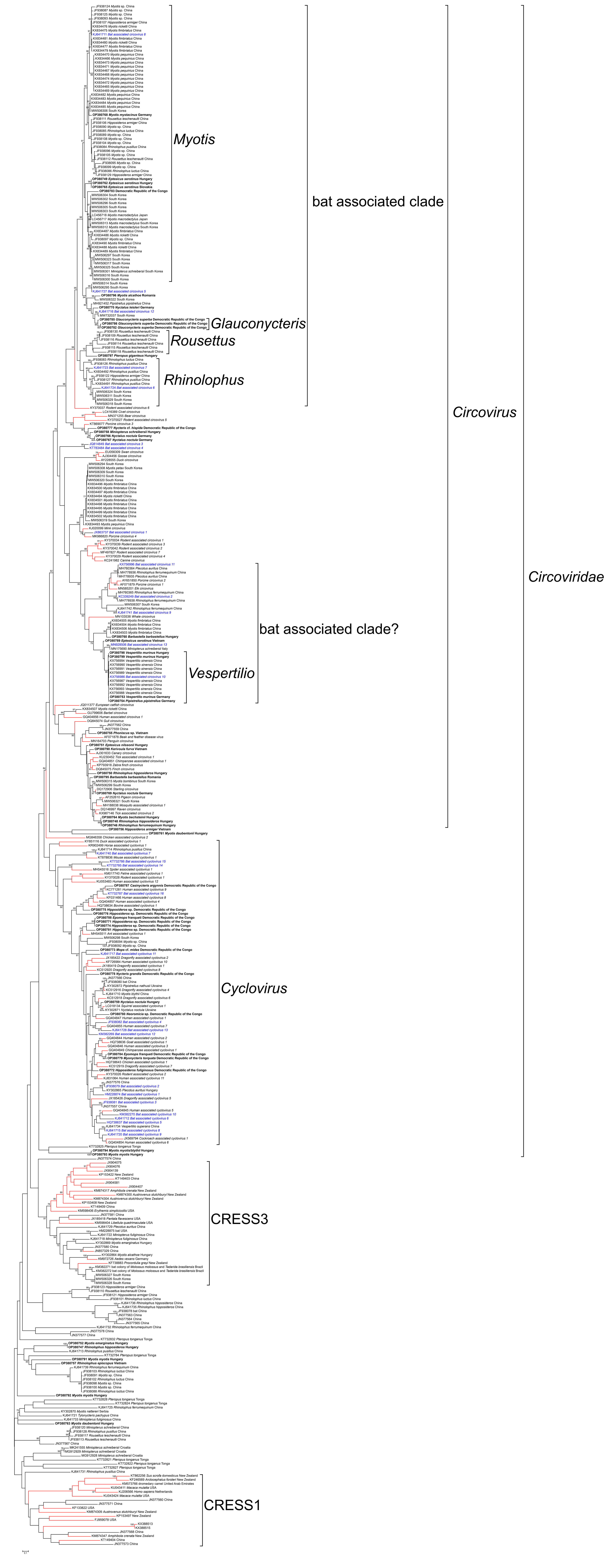

Myotis
bat associated clade
Glauconycteris
Rousettus
Rhinolophus
Circovirus
Circoviridae
bat associated clade?
Vespertilio
Cyclovirus
CRESS3
CRESS1
