# Supplementary material for: Detection and genetic characterization of circoviruses in more than 80 bat species from eight countries on four continents
Source: Vet Res Commun. 2023 Mar 31;47(3):1561–73. doi: 10.1007/s11259-023-10111-3 (PMC10066014; doi:10.1007/s11259-023-10111-3)
Supplement: Supplementary file 2 — Supplementary file2 (PDF 113 KB) Fig. S2 Phylogenetic tree reconstruction of bat cirliviruses with arfiviruses (class Arfiviricetes) based on Rep amino acid sequences. The strains are indicated by their nucleotide accession number, host species and country of collection (if available), except for CRESS1-5 strains, which are indicated by either nucleotide or Rep protein accession number. Newly detected virus strains are in bold, and branches of cirliviruses not associated with bats are indicated in red. The reference strains of bat associated circoviruses and cycloviruses are indicated in blue [file 11259_2023_10111_MOESM2_ESM.pdf]

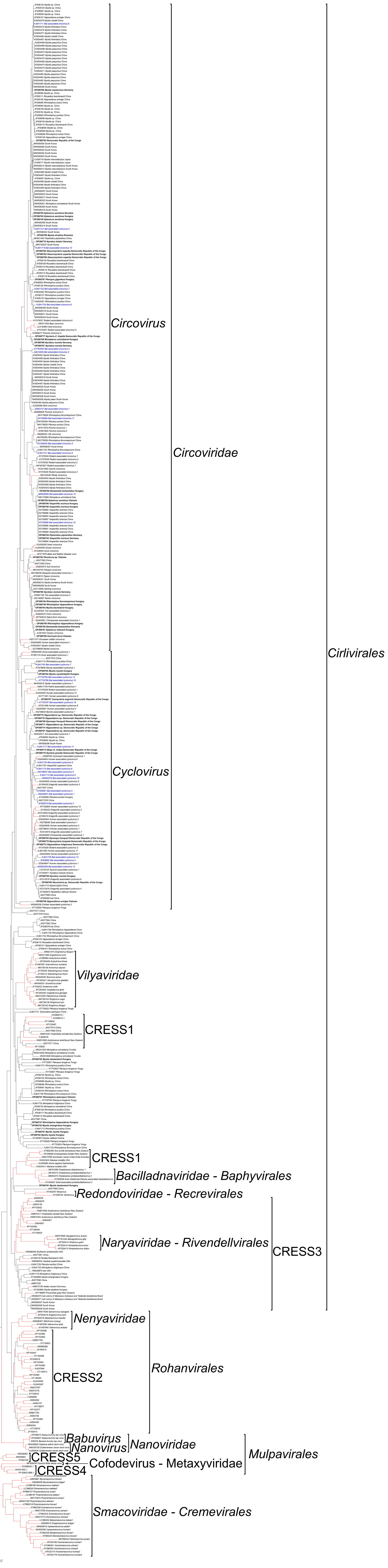
Circovirus
Circoviridae
Cirlivirales
Cyclovirus
Vilyaviridae
CRESS1
CRESS1
Bacilladnaviridae - Baphyvirales
Redondoviridae - Recrevirales
Naryaviridae - Rivendellvirales
CRESS3
Nenyaviridae
Rohanvirales
CRESS2
Babuvirus
Nanovirus
Nanoviridae
Mulpavirales
CRESS5
Cofodevirus - Metaxyviridae
CRESS4
Smacoviridae - Cremevirales
