# Supplementary material for: Detection and genetic characterization of circoviruses in more than 80 bat species from eight countries on four continents
Source: Vet Res Commun. 2023 Mar 31;47(3):1561–73. doi: 10.1007/s11259-023-10111-3 (PMC10066014; doi:10.1007/s11259-023-10111-3)
Supplement: Supplementary file 3 — Supplementary file3 (DOCX 13 KB) [file 11259_2023_10111_MOESM3_ESM.docx]

**Table S1.** Primers used for the sequencing of strain GT757B

| Reverse PCR primers | PCR F | CGGTATGCAAAGCAAGAGG |
| --- | --- | --- |
|  | PCR R | TTTAAAGCACGGCTCACCTT |
| Primers for primer walking | F2 | TGTGTGTTCTGTGTCTTTTTCC |
|  | R2 | TTTAGCCATGGCCCGTTG |
|  | R3 | CTAGGCCCGGGTTCAAAT |
